# Species Divergence and Phylogenetic Variation of Ecophysiological Traits in Lianas and Trees

**DOI:** 10.1371/journal.pone.0099871

**Published:** 2014-06-10

**Authors:** Rodrigo S. Rios, Cristian Salgado-Luarte, Ernesto Gianoli

**Affiliations:** 1 Departamento de Biología, Universidad de La Serena, La Serena, Chile; 2 Departamento de Botánica, Universidad de Concepción, Concepción, Chile; INRA - University of Bordeaux, France

## Abstract

The climbing habit is an evolutionary key innovation in plants because it is associated with enhanced clade diversification. We tested whether patterns of species divergence and variation of three ecophysiological traits that are fundamental for plant adaptation to light environments (maximum photosynthetic rate [A_max_], dark respiration rate [R_d_], and specific leaf area [SLA]) are consistent with this key innovation. Using data reported from four tropical forests and three temperate forests, we compared phylogenetic distance among species as well as the evolutionary rate, phylogenetic distance and phylogenetic signal of those traits in lianas and trees. Estimates of evolutionary rates showed that R_d_ evolved faster in lianas, while SLA evolved faster in trees. The mean phylogenetic distance was 1.2 times greater among liana species than among tree species. Likewise, estimates of phylogenetic distance indicated that lianas were less related than by chance alone (phylogenetic evenness across 63 species), and trees were more related than expected by chance (phylogenetic clustering across 71 species). Lianas showed evenness for R_d_, while trees showed phylogenetic clustering for this trait. In contrast, for SLA, lianas exhibited phylogenetic clustering and trees showed phylogenetic evenness. Lianas and trees showed patterns of ecophysiological trait variation among species that were independent of phylogenetic relatedness. We found support for the expected pattern of greater species divergence in lianas, but did not find consistent patterns regarding ecophysiological trait evolution and divergence. R_d_ followed the species-level pattern, i.e., greater divergence/evolution in lianas compared to trees, while the opposite occurred for SLA and no pattern was detected for A_max_. R_d_ may have driven lianas' divergence across forest environments, and might contribute to diversification in climber clades.

## Introduction

Climbing plants, in particular woody vines (lianas), are a distinctive component of mature forests in both tropical and temperate regions [1]–[3]. Data from long-term plots indicate that the dominance of lianas relative to trees is increasing in tropical forests [4], [5]. Moreover, liana abundance is negatively associated with tree carbon storage in tropical forests [6], [7]. The climbing habit has independently arisen numerous times throughout plant evolution [1], [8], and it seems to be a key innovation in angiosperms: climbing plant lineages have greater species richness than their non-climbing sister groups [9]. Thus, evidence from both ecological and macroevolutionary patterns suggests a performance advantage of lianas over trees.

Explanatory factors for the increased abundance and biomass of lianas in tropical forests include increasing forest disturbance, which increases local resource availability, and rising levels of atmospheric CO_2_ [4]. Moreover, increased abundance of lianas in seasonal forests during the dry season, as compared to trees, has been related to their increased efficiency in water uptake and transport, and higher photosynthetic rates ([10]–[12]; but see [13]). Thus, data suggest that lianas are better than trees at exploiting resource pulses. When providing functional arguments for the key innovation of the climbing habit (*sensu* [14]), Gianoli [9] suggested that ecological specialization may arise as a consequence of an hypothetically expanded light niche of lianas in the forest, which would result from the co-occurrence of unsupported (creeping) and supported (climbing) individuals that go up and down the forest canopy. This would maximize interactions with a wide array of antagonistic and mutualistic species [15], [16] that, in turn, might promote diversification [17]. It is increasingly recognized that purported evolutionary key innovations may be tested at an ecological time scale [14], [18]–[20].

Ecophysiological traits are fundamental components of plant adaptation to the environment [21], [22]. Specifically, A_max_ (maximum photosynthetic rate), R_d_ (dark respiration rate) and SLA (specific leaf area) play a key role in the phenotypic adjustment to heterogeneous light environments in both lianas and trees [23]–[26]. Thus, they reflect the balance between carbon gain (A_max_) and carbon use (R_d_), and the allocation of leaf biomass to light interception (SLA), which together determine plant growth and performance across light environments [27], [28]. Importantly, variation in plant functional traits observed at the population level is likely to be paralleled by evolutionary divergences under contrasting environments [29]. Moreover, the analysis of the phylogenetic structure of communities can provide insights to our understanding of trait evolution [30]. Recent studies have addressed phylogenetic variation in ecophysiological traits in climbing plants and trees [31]–[34], but their approach has been either exploratory (aiming to report global patterns) or methodological (testing new analytical tools); to our knowledge, a hypothesis-driven analysis is wanting.

Using data reported for several liana and tree species coexisting in tropical and temperate forests, and focusing on three key ecophysiological traits involved in plant adaptation across forest light gradients: A_max_, R_d_ and SLA, we herein compare lianas and trees in terms of trait evolutionary rates, phylogenetic diversity, phylogenetic trait diversity, and the phylogenetic signal. Thus, we compared the rate at which variance in the traits is accumulated among species per unit time at the tips of the phylogenetic tree [35], [36]. We also evaluated how similar is the average pair of species of lianas and trees both in terms of mean phylogenetic distance and trait variation [37]. We finally evaluated in lianas and trees the tendency for phylogenetically related species to resemble each other, i.e., the phylogenetic signal [38]. We tested the hypotheses that if the climbing habitat enhances clade diversification [9], and ecological divergence is the process underlying this pattern, then lianas should show higher trait evolutionary rates and greater species and trait divergence than trees under common environmental scenarios.

## Materials and Methods

### Data collection

We searched the literature for field studies in forest ecosystems where lianas and trees were analyzed for at least one of three ecophysiological traits: A_max_ on an area basis, R_d_, and SLA. We only chose those studies carried out in mature forests, where light heterogeneity across microsites is the greatest [3]. We only included native species because they have a long history of adaptation to the environment. We focused on angiosperms because of the availability of tools to reconstruct their phylogenetic history and estimate trait evolution (see below). The final data set included a pool of 63 liana species and 71 tree species belonging to four tropical forests (Gamboa, Panama; San Lorenzo, Panama; Riberalta, Bolivia; Xishuangbanna, China) and three temperate forests (Yakushidake, Japan; Beltsville, USA; Puyehue, Chile). We pooled species from all sites into growth forms, thus we had one liana “super-community” and one tree “super-community”. Phylogenetic analyses were conducted on these super-communities (see below). Detailed information, including study species, traits, sites, and data sources, is available in Supporting Information S1.

### Phylogeny reconstruction

**Figure 1:**
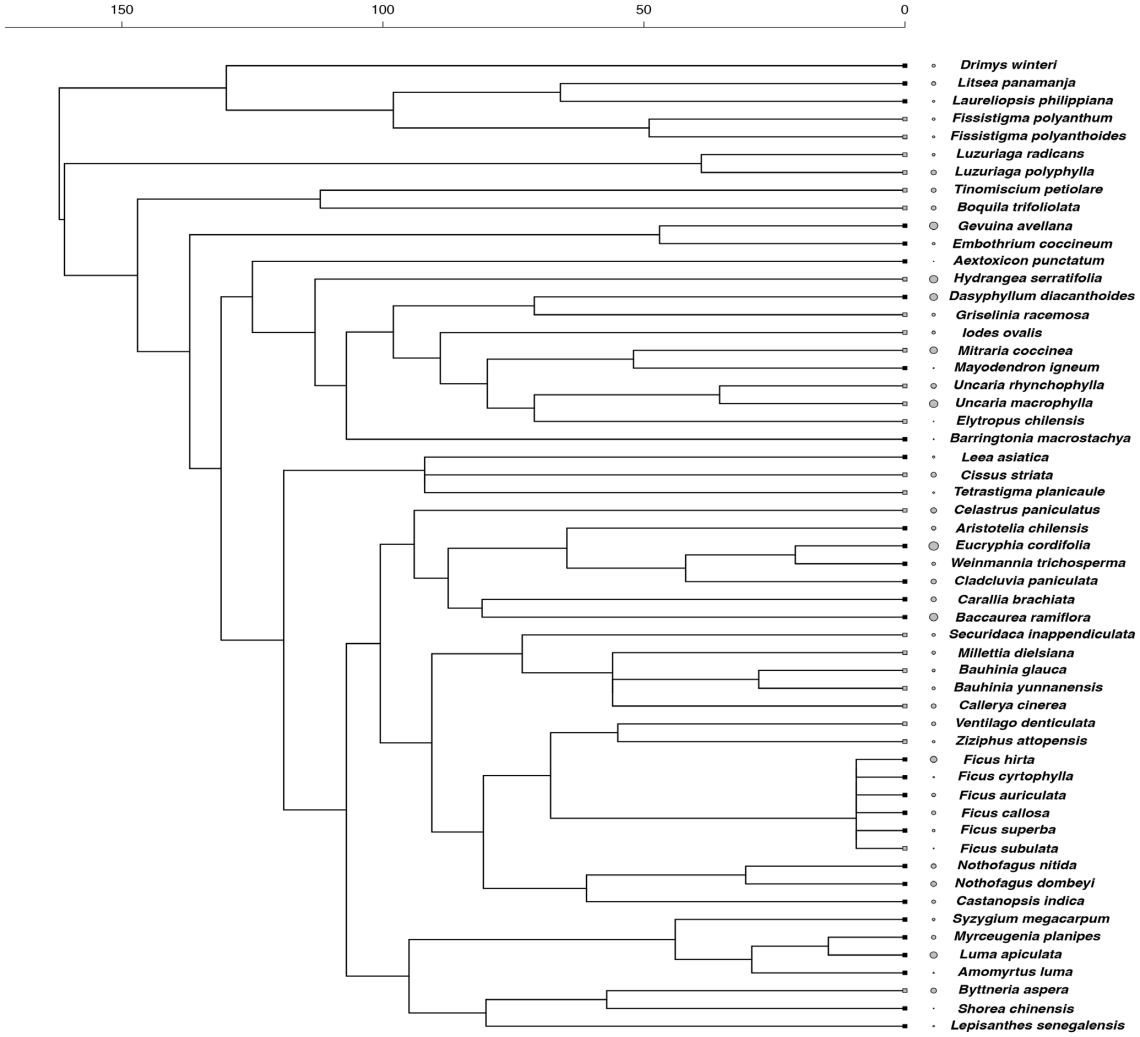
Phylogenetic relationships among tree and liana species and species values of dark respiration rate (R_d_). Grey circle size represents the proportional magnitude of the trait across species. Square tip symbols represent climbing habit (grey squares  =  liana, black squares  =  tree). Timescale is in millions of years before present.

**Figure 2:**
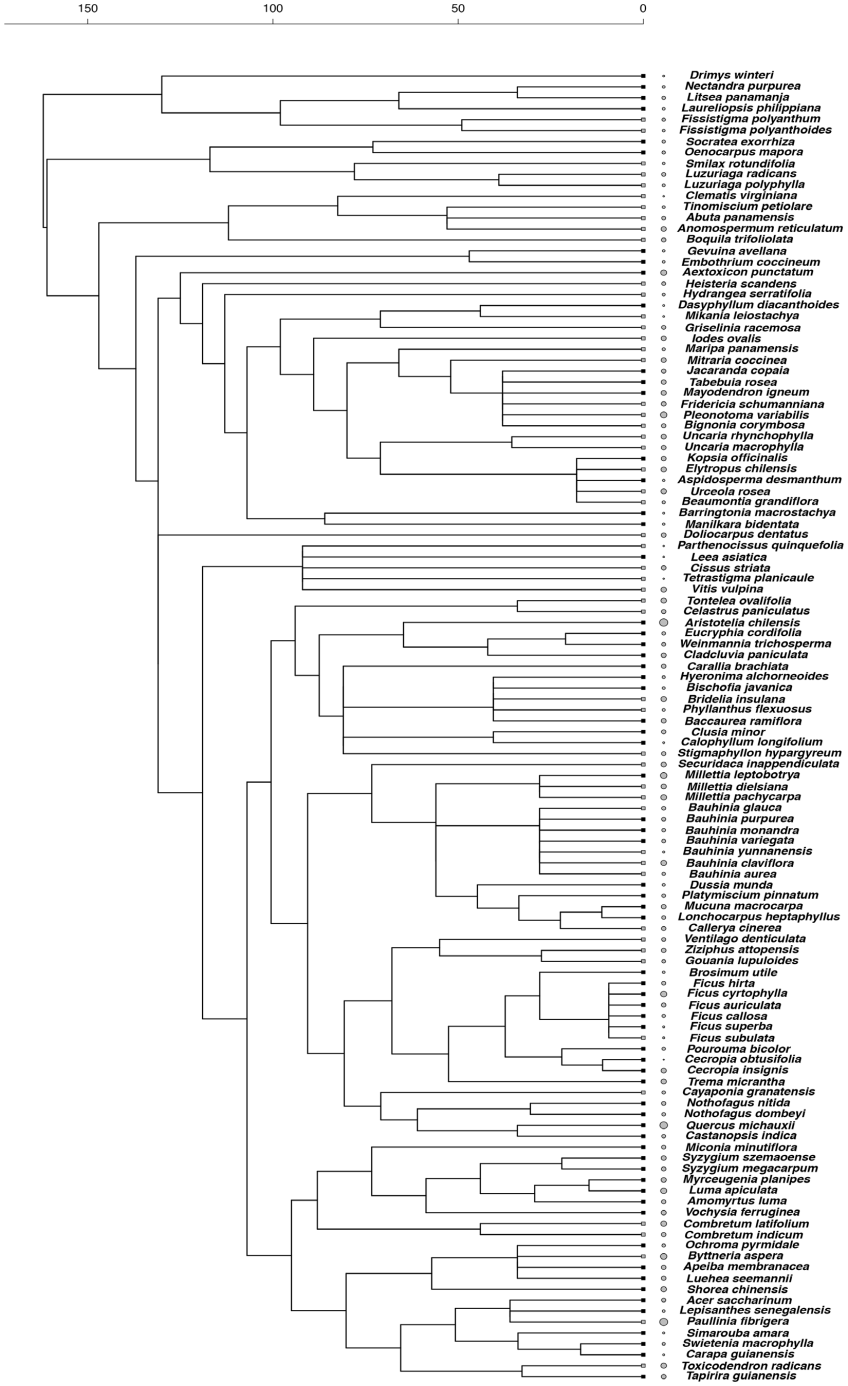
Phylogenetic relationships among tree and liana species and species values of maximum photosynthetic rate (A_max_). Grey circle size represents the proportional magnitude of the trait across species. Square tip symbols represent climbing habit (grey squares  =  liana, black squares  =  tree). Timescale is in millions of years before present.

**Figure 3:**
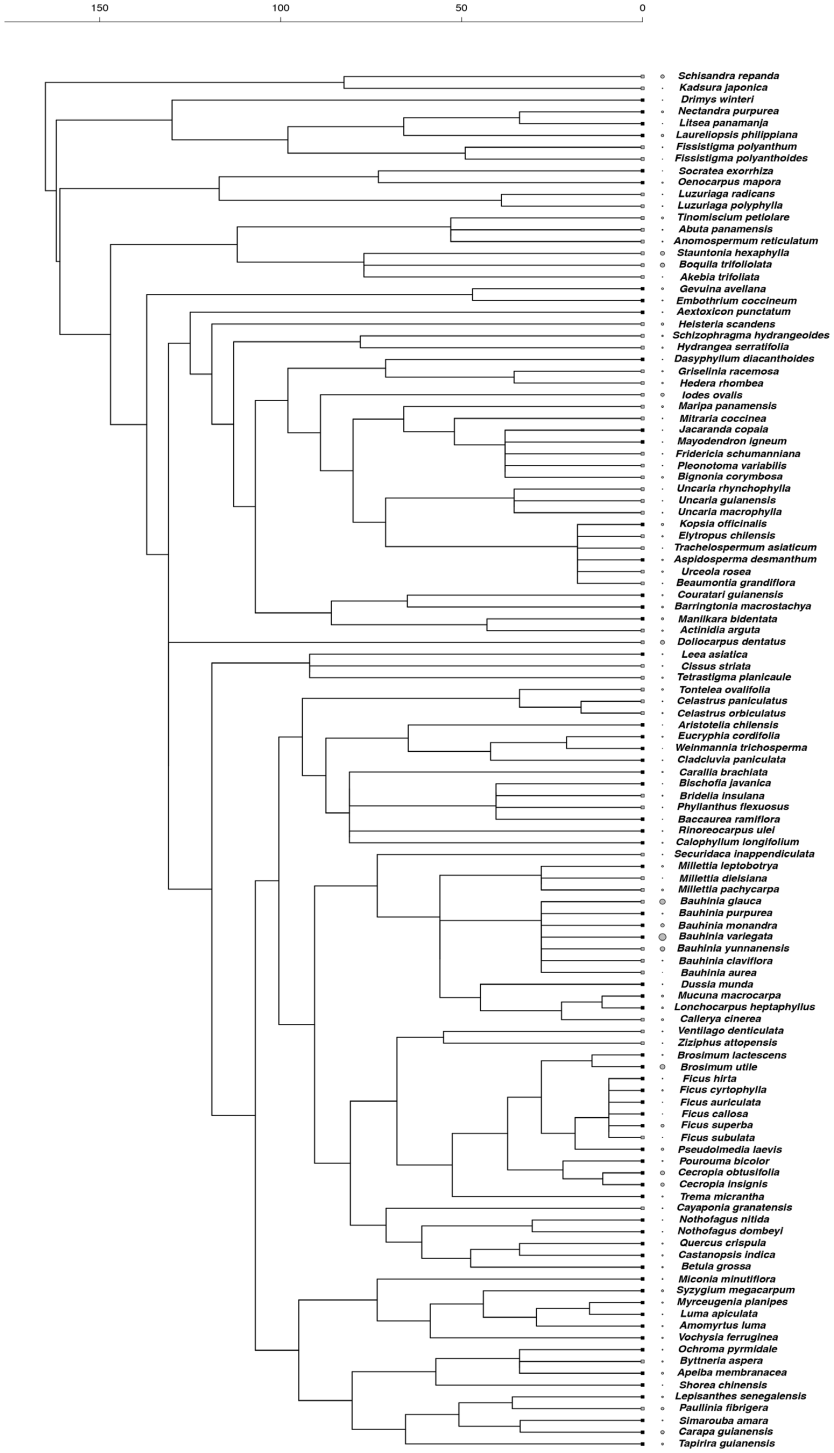
Phylogenetic relationships among tree and liana species and species values of specific leaf area (SLA). Grey circle size represents the proportional magnitude of the trait across species. Square tip symbols represent climbing habit (grey squares  =  liana, black squares  =  tree). Timescale is in millions of years before present.

We produced a phylogeny of all species using a backbone tree based on the angiosperm megatree provided by the Phylodiversity Network in cooperation with the Angiosperm Phylogeny Group (APG; http://www.mobot.org/MOBOT/research/APweb/). Our tree was generated using Phylomatic (http://www.phylodiversity.net/phylomatic/phylomatic.html), a program that returns a working phylogenetic tree after matching the genus and family names of study species to those contained in the angiosperm phylogeny [39]. Comparative inferences require branch lengths for the tree, which were calculated based on the branch length adjustment algorithm (BLADJ) implemented in Phylocom v. 4.2 (www.phylodiversity.net/phylocom) [40]. This algorithm fixes a subset of nodes in the tree to specified ages and evenly distributes the ages to the remaining nodes. Age estimates for major nodes in our tree were taken from [41]. To avoid inaccuracies in tree calibration and to have an updated version of our tree, we corrected the *ages* file with age estimates in [41] included in Phylocom. Corrections followed procedures suggested recently [42]. We also checked and updated age estimates of internal order-level clades according to a net diversification rate estimate of angiosperms [43]. The few polytomies in the working tree were resolved randomly using the *multi2di* function in R. Values of functional traits of closely related species resulting from such random resolutions were very similar, so results of the final comparative tests were highly robust to topological uncertainty. These and all subsequent analyses were conducted using the R statistical environment version 3.0.2 [44]. Reconstructed phylogenetic trees with associated trait variation are shown in Figures 1–3.

### Rate of trait evolution

To assess differences in ecophysiological trait evolution between liana and tree species, we compared estimates of evolutionary rate for A_max_, R_d_, and SLA. To this end, we first used stochastic character mapping, a Bayesian method that uses Monte Carlo simulations to sample the posterior probability distribution of ancestral states and timings of transitions on phylogenetic branches under a Markov process of evolution [45], [46]. We built stochastic character-mapped reconstructions for each trait/growth form combination using the *make.simmap* function in the phytools package of R [36]. We thus simulated character history evolution of all three traits in relation to growth form as an initial step, as suggested recently [35], [47]. To test the hypothesis that a discrete character state had influenced the rate of a continuous character, one should first stochastically map the discrete trait (e.g., climbing habit), and then test if one state of the discrete character has a different evolutionary rate for the continuous trait of interest (e.g., ecophysiological trait) than the other discrete state [35], [47].

The resulting reconstructions of trait states and phylogeny represented a set of phylogenetic topologies, branch lengths and growth forms sampled in proportion to their posterior probabilities. Reconstructions were then used in subsequent analyses as a way of integrating over uncertainty in phylogeny and ancestral states. Finally, we fitted the evolutionary models of character history on the trees to trait data using a likelihood method [35]. This is a maximum likelihood approach that estimates rates of evolution (σ^2^). The parameter σ^2^ was calculated using the function *brownie.lite* in the *phytools* package [36]. σ^2^ is interpreted as the Brownian motion process most likely to have produced the data at the tips of the tree, i.e., the rate at which variance is accumulated among species per unit time. 95% confidence intervals were calculated for each σ^2^ to infer differences between lianas and trees in ecophysiological trait evolution.

### Trait and phylogenetic diversity

To compare the phylogenetic relatedness among liana species against the phylogenetic relatedness among tree species we used measures of phylogenetic structure. Specifically, we calculated, based on a phylogenetic distance matrix, the mean phylogenetic distance (MPD) and the standardized effect size of the mean phylogenetic distance (SES_MPD_ [48]) between pairs of species for each group. Interspecific phylogenetic distance matrices were obtained from the reconstructed tree of phylogenetic relationships among taxa using the *cophenetic* function in R and unweighted pair-group average (UPGMA) as the clustering method.

Standardized effect sizes describe the difference between average phylogenetic distances in the observed super communities or groups (lianas and trees) compared to null distributions generated for each group with randomization procedures, standardized by the standard deviation of phylogenetic distances in the null data [48]. We compared observed mean distances (branch length) against a null model generated by calculating 999 times the mean phylogenetic distance between 8911 random pairs of species (without replacement) drawn from the matrix of phylogenetic distances between all liana and tree species. In all cases 999 iterations were found to be suitable for our randomization procedures as they were sufficient to attain convergence. The null model was constructed by reshuffling the distance of species labels across the phylogenetic tree using the *ses.mpd* function and the *taxa.labels* algorithm of the *picante* package of R. Positive values of SES_MPD_ (*mpd.obs.z*) and high quantiles (*p*-values >0.95) indicate significant phylogenetic evenness, while negative values of SES_MPD_ and low quantiles (*p*-values <0.05) indicate significant phylogenetic clustering [48]; these outcomes correspond to scenarios where species are more distantly or more closely related than expected by chance, respectively [48], [49]. Authors often refer to (weak) evenness or clustering when *p*-values are slightly lower than 0.95 or slightly higher than 0.05, respectively (e.g., [50], [51]). Finally, to assess how similar are the average pair of species within each group in terms of ecophysiological traits; we used the SES_MPD_ as a trait diversity measure. This was done by replacing the phylogenetic distance matrix in the analysis with a trait distance matrix, and proceeding accordingly to calculate standardized values of mean phylogenetic trait distance (SES_MTD_). These results are interpreted in the same way as those of SES_MPD_ with regard to phylogenetic evenness or clustering [48].

### Phylogenetic signal

To quantify the degree to which phylogenetic relatedness predicts the similarity of species in functional traits for both trees and lianas, we calculated separately phylogenetic signal for A_max_, R_d_, and SLA. Phylogenetic signal indicates to what extent phenotypic expression is explained by the lineage to which a species belongs, and it can be compared among clades and among traits [52]. We quantified phylogenetic signal using both Blomberg's *K* [38] and Pagel's *λ* [53] statistics for quantitative traits. To calculate these parameters, we first pruned two separate phylogenies, one for the group of lianas and one for the group of trees, using the original tree as a base phylogeny. Then we pruned a tree for each group-trait combination independently, removing taxa for which trait information was not available. The number of species included in each trait/plant growth habit analysis ranged from 26 (R_d_/lianas) to 67 (A_max_/trees), thus meeting the N>20 threshold to achieve good statistical power [38].

Values of *K* = 1 imply that a trait shows exactly the amount of phylogenetic signal expected under a null, stochastic model of character evolution (Brownian motion evolution) [38]. *K*-values >1 and <1 imply that close relatives are more similar and less similar, respectively, than expected under a Brownian motion model of trait evolution [38]. If *K* does not differ from zero it is concluded that the trait has no phylogenetic signal. Statistical significance of *K* [38] was assessed via permutation tests with 1000 randomizations. The significance of the phylogenetic signal was based on the variance of phylogenetically independent contrasts relative to tip shuffling randomization implemented by the *phylosignal* function of the *picante* package in R [48]. *P*-values were determined by comparing the variance of standardized independent contrasts for the tip values against variances for randomized data.

The parameter *λ* scales tree structure in terms of expected variances and covariances in trait change [54]. Thus, *λ* is a phylogenetic transformation that maximizes the likelihood of the data given a Brownian motion model [54]. When λ = 1, the trait is consistent with a Brownian motion evolution based on branch lengths represented by the variance-covariance in trait change. Values between 0 and 1 indicate less phylogenetic signal than expected under a Brownian motion model, while values >1 indicate more signal than expected, although λ is not always defined for values greater than one [54]. Values of λ were estimated using the *fitContinuous* function of the *geiger* package. To determine the significance of λ as an indicator of phylogenetic signal, we compared the maximum likelihood estimate of *λ* against the maximum likelihood of models when λ = 1 using likelihood ratio tests (LRT).

## Results and Discussion

### Rate of trait evolution

In general, lianas and trees presented homogenous evolution of ecophysiological traits. In all cases evolutionary rates, as estimated by σ^2^, were not significantly different from a single-rate Brownian motion process of evolution (Table 1). Parameter estimate values of σ^2^, however, did differ between lianas and trees in two of the three ecophysiological traits considered (Table 1). The evolutionary rate for dark respiration rate (R_d_) in lianas was 1.8 times greater than in trees. In the case of the biomass allocation trait (specific leaf area, SLA), the evolutionary rate was 1.2 times greater in trees than in lianas. Evolutionary rates for maximum photosynthetic rate (A_max_) did not differ between lianas and trees; overall, this trait showed the lowest evolutionary rate among the traits considered (Table 1).

**Table 1:** Parameter estimates and 95% confidence intervals (CI) of the evolutionary rate (σ^2^) for ecophysiological traits in lianas and trees.

	Lianas				Trees			
Trait	σ^2^	95% CI	*χ^2^*	*p*-value	σ^2^	95% CI	*χ^2^*	*p*-value
A_max_	0.075	0.072–0.079	−6.8	0.948	0.075	0.072–0.078	−10.6	0.995
R_d_	**0.148**	0.132–0.165	−4.1	0.999	**0.083**	0.076–0.091	−13.7	0.991
SLA	**0.093**	0.087–0.097	−11.8	0.999	**0.103**	0.099–0.108	−11.2	0.999

A_max_  =  maximum photosynthetic rate; R_d_  =  dark respiration rate; SLA  =  specific leaf area. Values of σ^2^ represent rates at which variance accumulates among species per unit time through a phylogeny with branch lengths in units of millions of years. *P*-values are for likelihood ratio tests against the chi-square distribution between a single-rate (homogenous) and a heterogeneous Brownian motion process. Bold cells indicate σ^2^ values for which 95% CI do not overlap between lianas and trees.

The patterns observed suggest that for all ecophysiological traits a change along any given branch in the phylogeny is independent of both previous changes and changes in other branches of the reconstructed tree. Evolutionary rates in both lianas and trees did not differ from a single-rate Brownian motion model of evolution, which assumes that variance among species in the phylogenetic tree accumulates as function of their time of independent evolution [55]. Thus, it cannot be ruled out that ecophysiological traits evolve at a constant rate over time. A Brownian motion process, however, is not equal to a neutral model of evolution. Brownian motion simply describes the distribution of observed trait changes and may be consistent with adaptive models of evolution [35], [56]. Therefore, natural selection could be a plausible force behind the alteration in rate change of traits in relation to growth form (climbers vs. non-climbers).

Evolutionary rates (σ^2^) differed between lianas and trees in two of the three ecophysiological traits considered, but in opposite trends. Thus, R_d_ evolved at a higher rate in lianas, while SLA evolution occurred at a higher rate in trees. This suggests that the outcome of modifications in the selective regime related to the climbing habit depends on the particular plant traits that are under selection (gas-exchange traits vs. biomass allocation traits). Gas-exchange traits have been shown to be of selective value for the exploitation of light availability in mature forests for trees [57], vines [58] and ferns [59]. Our findings suggest that climbers are more evolutionary responsive with regard to R_d_ than trees. Assuming that (adaptive) ecological speciation is the process behind species divergence in this trait [20], the next step would be to address whether this results from a greater magnitude of selection on R_d_ or from greater trait heritability [21], [60]. Conversely, SLA showed a higher greater evolutionary rate across tree species. This somewhat supports the view of SLA as an essential attribute for tree performance and carbon gain [57], [61].

### Trait and phylogenetic diversity

We found that mean phylogenetic distance (MPD, non-standardized values) was greater among liana species (259.9 Myr) than among tree species (229.6 Myr). Moreover, there was a clear-cut difference between lianas and trees in the standardized mean phylogenetic distance among species (SES_MPD_). Whereas lianas showed greater distances between species relative to the null model (SES_MPD_ = 2.271; *p*-value  = 0.99), i.e., phylogenetic evenness, trees showed a pattern of phylogenetic clustering (SES_MPD_  = −3.622; *p*-value  = 0.006).

Lianas and trees differed in their patterns of trait diversity. For one of the gas-exchange traits (R_d_), lianas showed phylogenetic evenness (SES_MTD_  = 1.266, *p*-value  = 0.893), which means that trait dissimilarity among liana species was higher than expected by chance, while trees showed phylogenetic clustering (SES_MTD_  = −1.863, *p*-value  = 0.039), indicating that tree species were more phenotypically similar than expected by chance (Figure 1). In contrast, for the biomass allocation trait (SLA), lianas exhibited phylogenetic clustering (SES_MTD_  = −1.194, *p*-value  = 0.122) and trees showed phylogenetic evenness (SES_MTD_  = 1.193, *p*-value  = 0.877) (Figure 2). Finally, the other gas-exchange trait, A_max_, did not show phylogenetic structure in both lianas (SES_MTD_  = 0.096, *p*-value  = 0.536) and trees (SES_MTD_  = −0.598, *p*-value  = 0.277) (Figure 3).

First, in agreement with the study hypotheses, we found greater phylogenetic distance among species within the group of lianas (63 species) than within the group of trees (71 species). This agrees with a recent study in Australian rainforests, where standardized values of mean phylogenetic distance indicated that climbers show weak evenness or no phylogenetic structure, while trees/shrubs show weak to significant phylogenetic clustering [50]. Second, the average phenotypic distance among species for one gas-exchange trait (but not for the other two ecophysiological traits) was greater in the phylogenetic tree of lianas than in that of trees. The environmental gradient experienced by the study species was roughly the same for lianas and trees because data were obtained from sites where trees and lianas coexisted. Therefore, these patterns of (partial) increased phenotypic distance and greater phylogenetic divergence in lianas are consistent with the notion that lianas have a greater differentiation potential than trees [9]. Several plant attributes have been associated with evolutionary rates in angiosperms. For instance, it has been shown that trees and shrubs have lower rates of molecular evolution than herbaceous plants [62], [63], and that taller plants have lower rates of molecular evolution [64]; in both cases the outcome is thought to be linked to differences in generation time, which in turn is related to mutation rate. In our study all climbers were woody species (lianas) so herbaceousness should not be a confounded factor. However, there is no available information to reject the possibility that there were longer generation times in the group of trees (see General Conclusions). As to the plant height factor, it is a rather problematic issue, because trees are usually taller than lianas in terms of freestanding height, but if total length is considered, then canopy lianas may be taller. Both issues deserve further scrutiny.

In the realm of community phylogenetics, patterns of phylogenetic evenness in resource-use traits are often interpreted to reflect niche differentiation processes [49]. If trait-based niche differentiation facilitates evolutionary responses to divergent selection, which in turn may lead to incipient speciation [65], then our results of phylogenetic evenness in a liana ecophysiological trait might be linked to the ecological/evolutionary processes that underlie the key innovation of the climbing habit in plants [9]. Whereas results of trait phylogenetic distance suggest that R_d_ may have played a role as driver of lianas' adaptive divergence, SLA showed greater phenotypic divergence among tree species, as was shown for rates of trait evolution (see above). However, this trait distribution pattern across the phylogenetic tree was not accompanied by an overall greater phylogenetic distance among tree species. This might be interpreted as SLA contributing to tree adaptation to environmental challenges at local scales but do not driving taxonomic divergence across clades.

### Phylogenetic signal

Overall, lianas and trees presented mixed but comparable patterns of phylogenetic signal (or lack thereof) in ecophysiological traits (Table 2). In lianas, A_max_ showed no significant phylogenetic signal, but values were lower than expected under a Brownian model of evolution (with *K*<1 and *λ*<1). R_d_ showed mixed results, with significant phylogenetic signal indicated by *K*, and lower than expected under a Brownian model of evolution, but no significant signal as indicated by *λ* ( = 1). There was no phylogenetic signal detected for specific leaf area (SLA) using *K* but strong signal using *λ*, and lower than expected under a Brownian model of evolution (Table 2). In trees, whereas no phylogenetic signal was found for both A_max_ and R_d_ as indicated by *K* and *λ*, a significant phylogenetic signal was found for SLA when *K* is considered (with *K*<1) but no signal was detected by *λ*, with values lower than expected under a Brownian model of trait evolution (*λ*≈0) (Table 2). Summarizing, in all cases both lianas and trees tended to show patterns of ecophysiological trait variation among species that were independent of phylogenetic relatedness.

**Table 2:** Phylogenetic signal, quantified as Blomberg's *K* and Pagel's λ, for three ecophysiological traits in lianas and trees.

	Lianas				Trees			
Trait	*K*	*p*-value	λ	*p*-value	*K*	*p*-value	λ	*p*-value
A_max_	0.518	0.157	0.338	0.281	0.308	0.459	0.223	0.503
R_d_	**0.874**	0.015	1.000	0.096	0.284	0.573	0.125	0.713
SLA	0.511	0.166	**<0.001**	<0.001	**0.638**	0.001	0.096	0.282

A_max_  =  maximum photosynthetic rate; R_d_  =  dark respiration rate; SLA  =  specific leaf area.

Significant phylogenetic signals are shown in bold.

Our results are consistent with the general pattern that physiological traits tend to show low values of phylogenetic signal [38]. A global analysis of trait variation in climbing plants reported that SLA showed no phylogenetic signal [34], as found in the present study. Likewise, in agreement with our results, a global-scale study in Angiosperms reported that A_max_ (on an area basis) showed no consistent phylogenetic signal [32]. Conversely, a genus-level study in trees [31] found that A_max_ exhibited significant phylogenetic signal, which seemingly opposes our findings. However, this study used a metric other than Blomberg's *K* and Pagel's *λ*, and given that different indices of phylogenetic signal often lead to contrasting outcomes ([68]; and Table 2), these results are not necessarily contrary to those reported here. Another group of ecophysiological traits that could have been studied to seek phylogenetic and evolutionary differences between trees and lianas is that of hydraulic characters. Regarding hydraulic traits, lianas have wider and longer vessels compared to trees, features that enable them to supply a large leaf area with a relatively small allocation to xylem tissue [33], [66], [67]. However, xylem vessel length did not show significant phylogenetic signal in a recent global analysis including lianas, shrubs and trees [33].

Results indicate that, in both lianas and trees, ecophysiological traits related to light use and carbon economy have undergone evolutionary trajectories different to those expected after phylogenetic relationships, assuming a Brownian motion model of trait evolution [68]. These phylogenetic signal results do not match the patterns of trait divergence and trait evolutionary rates found here. Although under some circumstances (e.g., fluctuating selection in related lineages) a negative association between *K* and evolutionary rate may be found [52], [55], it is generally considered that changes in trait evolutionary rates –and ensuing phenotypic divergence– does not influence phylogenetic signal for continuous characters [52], [55].

### General conclusions

Lianas and trees differ in a number of anatomical, physiological, morphological and life history traits [69]–[71]. Among the main differences, trees show a greater allocation of biomass (and carbon) to stems and lianas have lower costs of height gain and larger total leaf area potential. Moreover, compared to shrubs and trees, lianas have lower leaf mass per area (LMA, the inverse of SLA), higher foliar N and higher mass-based photosynthetic rate, which is consistent with the characterization of lianas as fast metabolism/rapid turnover species [71]. This could be related to hypothetical differences in generation time between lianas and trees that could explain their differential evolutionary rates, as shown here. Nonetheless, when it comes to explain species distribution across the light gradient in forests [72], the life history trade-off between juvenile growth and survival is observed alike in trees and lianas [73].

Phylogenetic information is increasingly used to test macroevolutionary hypotheses of trait evolution [74]–[76]. The study hypotheses, arising from the macroevolutionary pattern of increased taxonomic diversification in lianas [9], received mixed support. Overall, mean phylogenetic distance among liana species was larger than that of trees. Lianas showed a higher evolutionary rate for a gas-exchange trait (R_d_), but the biomass allocation trait (SLA) evolved at a higher rate in trees. Likewise, average trait divergence across the phylogenetic tree was greater in lianas for R_d_ but it was greater in trees for SLA. Therefore, although we have found support for the expected pattern of increased species divergence in lianas compared to trees, we did not find consistent patterns regarding ecophysiological trait evolution and divergence. R_d_ followed the species-level patterns, i.e., greater divergence/evolution in lianas compared to trees, while the opposite was found for SLA. R_d_ may have driven lianas' divergence across forest environments and, furthermore, might contribute to the pattern of increased diversification in climber clades.

## Supporting Information

Supporting Information S1 **Study sites (forests), liana and tree species, ecophysiological traits, and source references.**
(XLS)

## References

[1] Gentry AH (1991) The distribution and evolution of climbing plants. In: Putz FE, Mooney HA, eds. The biology of vines. Cambridge: Cambridge University Press.pp 3–49.

[2] Schnitzer SA, Bongers F (2002) The ecology of lianas and their role in forests. Trends Ecol Evol 17: 223–230.

[3] Gianoli E, Saldaña A, Jiménez-Castillo M, Valladares F (2010) Distribution and abundance of vines along the light gradient in a southern temperate rainforest. J Veg Sci 21: 66–73.

[4] Schnitzer SA, Bongers F (2011) Increasing liana abundance and biomass in tropical forests: emerging patterns and putative mechanisms. Ecol Lett 14: 397–406. 21314879 10.1111/j.1461-0248.2011.01590.x

[5] Phillips OL, Martínez RV, Arroyo L, Baker TR, Killeen T, et al (2002) Increasing dominance of large lianas in Amazonian forests. Nature 418: 770–774. 12181565 10.1038/nature00926

[6] Durán SM, Gianoli E (2013) Carbon stocks in tropical forests decrease with liana density. Biol Lett 9: 20130301. 23784930 10.1098/rsbl.2013.0301 PMC3730642

[7] van der Heijden GMF, Schnitzer SA, Power JS, Phillips OL (2013) Liana impacts on carbon cycling, storage and sequestration in tropical forests. Biotropica 45: 682–692.

[8] Gianoli E (2014) Evolutionary implications of the climbing habit in plants. In: Schnitzer SA, Bongers F, Burnham RJ, Putz FE, eds. Ecology of lianas. New York: Wiley-Blackwell. *in press*.

[9] Gianoli E (2004) Evolution of a climbing habit promotes diversification in flowering plants. Proc R Soc Lond B 271: 2011–2015. 10.1098/rspb.2004.2827 PMC1691831 15451690

[10] Schnitzer SA (2005) A mechanistic explanation for global patterns of liana abundance and distribution. Am Nat 166: 262–276. 16032578 10.1086/431250

[11] Cai Z-Q, Schnitzer SA, Bongers F (2009) Seasonal differences in leaf-level physiology give lianas a competitive advantage over trees in a tropical seasonal forest. Oecologia 161: 25–33. 19418072 10.1007/s00442-009-1355-4 PMC2700874

[12] Zhu S-D, Cao K-F (2009) Hydraulic properties and photosynthetic rates in co-occurring lianas and trees in a seasonal tropical rainforest in southwestern China. Plant Ecol 204: 295–304.

[13] van der Sande MT, Poorter L, Schnitzer SA, Markesteijn L (2013) Are lianas more drought tolerant than trees? A test for the role of hydraulic architecture and other stem and leaf traits. Oecologia 172: 961–972. 23277211 10.1007/s00442-012-2563-x

[14] Heard SB, Hauser D (1995) Key evolutionary innovations and their ecological mechanisms. Hist Biol 10: 151–173.

[15] Gentry AH (1991) Breeding and dispersal systems of lianas. In: Putz FE, Mooney HA, eds. The biology of vines. Cambridge: Cambridge University Press.pp 393–423.

[16] Riveros M, Smith-Ramírez C (1995) Patrones de floración y fructificación en bosques del sur de Chile. In: Armesto JJ, Villagrán C, Arroyo MK, eds. Ecología de los bosques nativos de Chile. Santiago: Editorial Universitaria. pp. 235–250.

[17] Futuyma DJ, Agrawal AA (2009) Macroevolution and the biological diversity of plants and herbivores. Proc Natl Acad Sci USA 106: 18054–18061. 19815508 10.1073/pnas.0904106106 PMC2775342

[18] Hunter JP (1998) Key innovations and the ecology of macroevolution. Trends Ecol Evol 13: 31–36. 21238187 10.1016/s0169-5347(97)01273-1

[19] Schluter D (2000) The ecology of adaptive radiation. Oxford: Oxford University Press. 288 p.

[20] Funk DJ, Nosil P, Etges WJ (2006) Ecological divergence exhibits consistently positive associations with reproductive isolation across disparate taxa. Proc Natl Acad Sci USA 103: 3209–3213. 16492742 10.1073/pnas.0508653103 PMC1413886

[21] Ackerly DD, Dudley SA, Sultan SE, Schmitt J, Coleman JS, et al (2000) The evolution of plant ecophysiological traits: recent advances and future directions. Bioscience 50: 979–995.

[22] Wright IJ, Reich PB, Westoby M, Ackerly DD, Baruch Z, et al (2004) The worldwide leaf economics spectrum. Nature 428: 821–827. 15103368 10.1038/nature02403

[23] Poorter L, Bongers F (2006) Leaf traits are good predictors of plant performance across 53 rain forest species. Ecology 87: 1733–1743. 16922323 10.1890/0012-9658(2006)87[1733:ltagpo]2.0.co;2

[24] Santiago LS, Wright SJ (2007) Leaf functional traits of tropical forest plants in relation to growth form. Functional Ecology 21: 19–27.

[25] Valladares F, Niinemets Ü (2008) Shade tolerance, a key plant feature of complex nature and consequences. Annu Rev Ecol Evol Syst 39: 237–257.

[26] Gianoli E, Saldaña A, Jiménez-Castillo M (2012) Ecophysiological traits may explain the abundance of climbing plant species across the light gradient in a temperate rainforest. PLoS ONE 7(6): e38831. 22685611 10.1371/journal.pone.0038831 PMC3369858

[27] Lambers H, Chapin FS, Pons TL (1998) Growth and allocation. In: Lambers H, Chapin FS, Pons TL, eds. Plant physiological ecology. New York: Springer. pp. 299–351.

[28] Pearcy RW (2007) Responses of plants to heterogeneous light environments. In: Pugnaire FI, Valladares F, eds. Functional plant ecology. Boca Raton: CRC Press. pp. 213–257.

[29] Ackerly DD (1999) Comparative plant ecology and the role of phylogenetic information. In: Press MC, Scholes JD, Barker MG, eds. Physiological plant ecology. Oxford: Blackwell Science.pp 391–413.

[30] Vamosi SM, Heard SB, Vamosi JC, Webb CO (2009) Emerging patterns in the comparative analysis of phylogenetic community structure. Mol Ecol 18: 572–592. 19037898 10.1111/j.1365-294X.2008.04001.x

[31] Zheng L, Ives AR, Garland T, Larget BR, Yu Y, et al (2009) New multivariate tests for phylogenetic signal and trait correlations applied to ecophysiological phenotypes of nine *Manglietia* species. Funct Ecol 23: 1059–1069.

[32] Walls RL (2011) Angiosperm leaf vein patterns are linked to leaf functions in a global-scale data set. Am J Bot 98: 244–253. 21613113 10.3732/ajb.1000154

[33] Jacobsen AL, Pratt RB, Tobin MF, Hacke UG, Ewers FW (2012) A global analysis of xylem vessel length in woody plants. Am J Bot 99: 1583–1591. 22965850 10.3732/ajb.1200140

[34] Gallagher RV, Leishman MR (2012) A global analysis of trait variation and evolution in climbing plants. J Biogeogr 39: 1757–1771.

[35] O'Meara BC, Ané C, Sanderson MJ, Wainwright PC (2006) Testing for different rates of continuous trait evolution using likelihood. Evolution 60: 922–933. 16817533

[36] Revell LJ (2012) phytools: an R package for phylogenetic comparative biology (and other things). Methods Ecol Evol 3: 217–223.

[37] Webb CO, Ackerly DD, McPeek MA, Donoghue MJ (2002) Phylogenies and community ecology. Annu Rev Ecol Syst 33: 475–505.

[38] Blomberg SP, Garland T, Ives AR (2003) Testing for phylogenetic signal in comparative data: behavioral traits are more labile. Evolution 57: 717–745. 12778543 10.1111/j.0014-3820.2003.tb00285.x

[39] Webb CO, Donoghue MJ (2005) Phylomatic: tree assembly for applied phylogenetics. Mol Ecol Notes 5: 181–183.

[40] Webb CO, Ackerly DD, Kembel SW (2008) Phylocom: software for the analysis of phylogenetic community structure and trait evolution. Bioinformatics 24: 2098–2100. 18678590 10.1093/bioinformatics/btn358

[41] Wikstrom N, Savolainen V, Chase MW (2001) Evolution of the angiosperms: calibrating the family tree. Proc R Soc Lond B 268: 2211–2220. 10.1098/rspb.2001.1782 PMC1088868 11674868

[42] Gastauer M, Meira-Neto JAA (2013) Avoiding inaccuracies in tree calibration and phylogenetic community analysis using Phylocom 4.2. Ecol Inform 15: 85–90.

[43] Magallon M, Castillo S (2009) Angiosperm diversification through time. Am J Bot 96: 349–365. 21628193 10.3732/ajb.0800060

[44] R Core Team (2013) R: A language and environment for statistical computing. R Foundation for Statistical Computing, Vienna, Austria. URL http://www.R-project.org/.

[45] Nielsen R (2002) Mapping mutations on phylogenies. Syst Biol 51: 729–739. 12396587 10.1080/10635150290102393

[46] Huelsenbeck JP, Nielsen R, Bollback JP (2003) Stochastic mapping of morphological characters. Syst Biol 52: 131–158. 12746144 10.1080/10635150390192780

[47] Revell LJ (2013) A comment on the use of stochastic character maps to estimate evolutionary rate variation in a continuously valued trait. Syst Biol 62: 339–345. 23027088 10.1093/sysbio/sys084

[48] Kembel SW, Cowan PD, Helmus MR, Cornwell WK, Morlon H, et al (2010) Picante: R tools for integrating phylogenies and ecology. Bioinformatics 26: 1463–1464. 20395285 10.1093/bioinformatics/btq166

[49] Kraft NJB, Ackerly DD (2010) Functional trait and phylogenetic tests of community assembly across spatial scales in an Amazonian forest. Ecol Monogr 80: 401–422.

[50] Kooyman RM, Rossetto M, Sauquet H, Laffan SW (2013) Landscape patterns in rainforest phylogenetic signal: isolated islands of refugia or structured continental distributions? PLoS ONE 8(12): e80685. 24312493 10.1371/journal.pone.0080685 PMC3846590

[51] Verdú M, Pausas JG (2007) Fire drives phylogenetic clustering in Mediterranean Basin woody plant communities. J Ecol 95: 1316–1323.

[52] Ackerly DD (2009) Conservatism and diversification of plant functional traits: evolutionary rates versus phylogenetic signal. Proc Natl Acad Sci USA 106: 19699–19706. 19843698 10.1073/pnas.0901635106 PMC2780941

[53] Pagel M (1999) Inferring the historical patterns of biological evolution. Nature 401: 877–884. 10553904 10.1038/44766

[54] Freckleton RP, Harvey PH, Pagel M (2002) Phylogenetic analysis and comparative data: a test and review of evidence. Am Nat 160: 712–726. 18707460 10.1086/343873

[55] Revell LJ, Harmon LJ, Collar DC (2008) Phylogenetic signal, evolutionary process, and rate. Syst Biol 57: 591–601. 18709597 10.1080/10635150802302427

[56] Hansen TF, Martins EF (1996) Translating between microevolutionary process and macroevolutionary patterns: The correlation structure of interspecific data. Evolution 50: 1404–14017. 28565714 10.1111/j.1558-5646.1996.tb03914.x

[57] Salgado-Luarte C, Gianoli E (2012) Herbivores modify selection on plant functional traits in a temperate rainforest understory. Am Nat 180: E42–E53. 22766937 10.1086/666612

[58] Gianoli E, Saldaña A (2013) Phenotypic selection on leaf functional traits of two congeneric species in a temperate rainforest is consistent with their shade tolerance. Oecologia 173: 13–21. 23334233 10.1007/s00442-013-2590-2

[59] Saldaña A, Lusk CH, Gonzáles WL, Gianoli E (2007) Natural selection on ecophysiological traits of a fern species in a temperate rainforest. Evol Ecol 21: 651–662.

[60] Geber MA, Griffen LR (2003) Inheritance and natural selection on functional traits. Int J Plant Sci 164: S21–S42.

[61] Evans JR, Poorter H (2001) Photosynthetic acclimation of plants to growth irradiance: the relative importance of specific leaf area and nitrogen partitioning in maximizing carbon gain. Plant Cell Env 24: 755–767.

[62] Dodd ME, Silvertown J, Chase MW (1999) Phylogenetic analysis of trait evolution and species diversity variation among angiosperm families. Evolution 53: 732–744. 28565649 10.1111/j.1558-5646.1999.tb05367.x

[63] Smith SA, Donoghue MJ (2008) Rates of molecular evolution are linked to life history in flowering plants. Science 322: 86–89. 18832643 10.1126/science.1163197

[64] Lanfear R, Ho SY, Davies TJ, Moles AT, Aarssen L, et al (2013) Taller plants have lower rates of molecular evolution. Nature Comm 4: 1879. 10.1038/ncomms2836 23695673

[65] Nosil P, Harmon LJ, Seehausen O (2009) Ecological explanations for (incomplete) speciation. Trends Ecol Evol 24: 145–156. 19185951 10.1016/j.tree.2008.10.011

[66] Gartner BL, Bullock SH, Mooney HA, Brown VB, Whitbeck JL (1990) Water transport properties of vine and tree stems in a tropical deciduous forest. Am J Bot 77: 742–749.

[67] Ewers FW, Fisher JB (1991) Why vines have narrow stems: histological trends in *Bauhinia* . Oecologia 88: 233–237. 28312137 10.1007/BF00320816

[68] Münkemüller T, Lavergne S, Bzeznik B, Dray S, Jombart T, et al (2012) How to measure and test phylogenetic signal. Methods Ecol Evol 3: 743–756.

[69] Cornelissen JHC, Werger MJA, Castro-Diez P, van Rheenen JWA, Rowland AP (1997) Foliar nutrients in relation to growth, allocation and leaf traits in seedlings of a wide range of woody plant species and types. Oecologia 111: 460–469. 28308106 10.1007/s004420050259

[70] Cai ZQ, Schnitzer SA, Bongers F (2009) Seasonal differences in leaf-level physiology give lianas a competitive advantage over trees in a tropical seasonal forest. Oecologia 161: 25–33. 19418072 10.1007/s00442-009-1355-4 PMC2700874

[71] Wyka TP, Oleksyn J, Karolewski P, Schnitzer SA (2013) Phenotypic correlates of the lianescent growth form: a review. Ann Bot 112: 1667–1681. 24169592 10.1093/aob/mct236 PMC3838560

[72] Wright SJ (2002) Plant diversity in tropical forests: a review of mechanisms of species coexistence. Oecologia 130: 1–14. 28547014 10.1007/s004420100809

[73] Gilbert B, Wright SJ, Muller-Landau HC, Kitajima K, Hernández A (2006) Life history trade-offs in tropical trees and lianas. Ecology 87: 1281–1288. 16761606 10.1890/0012-9658(2006)87[1281:lhtitt]2.0.co;2

[74] Mooers AØ, Vamosi SM, Schluter D (1999) Using phylogenies to test macroevolutionary hypotheses of trait evolution in cranes (Gruinae). Am Nat 154: 249–259. 29578789 10.1086/303226

[75] Adams DC, Berns CM, Kozak KH, Wiens JJ (2009) Are rates of species diversification correlated with rates of morphological evolution? Proc R Soc B 276: 2729–2738. 10.1098/rspb.2009.0543 PMC2839962 19439441

[76] Magnuson-Ford K, Otto SP (2012) Linking the investigations of character evolution and species diversification. Am Nat 180: 225–245. 22766933 10.1086/666649

